# Telepractice Parent Training of Enhanced Milieu Teaching With Phonological Emphasis (EMT+PE) For Persian-Speaking Toddlers With Nonsyndromic Cleft Palate: Protocol for a Randomized Controlled Trial

**DOI:** 10.2196/54426

**Published:** 2024-04-19

**Authors:** Neda Tahmasebi, Talieh Zarifian, Atieh Ashtari, Akbar Biglarian

**Affiliations:** 1 Department of Speech Therapy Pediatric Neurorehabilitation Research Center University of Social Welfare and Rehabilitation Sciences Tehran Iran; 2 Department of Biostatistics and Epidemiology Social Determinants of Health Research Center, Social Health Research Institute University of Social Welfare and Rehabilitation Sciences Tehran Iran

**Keywords:** telepractice, cleft palate, language intervention, parent training, Phonological Emphasis, Enhanced Milieu Teaching, Persian-speaking toddlers, toddler, toddlers, children, child, cleft lip, language development, speech sound disorders, speech sound disorder, effectiveness, parent-based, intervention, speech, therapy

## Abstract

**Background:**

Children born with a cleft palate with or without a cleft lip (CP/L) are at increased risk for delayed language development and speech sound disorders. Enhanced Milieu Teaching with Phonological Emphasis (EMT+PE) is a recommended naturalistic intervention for toddlers with CP/L. The parents’ role in providing naturalistic interventions is critical and they need training based on learning principles to implement these interventions. Telepractice is an appropriate method for training parents and children with various speech-related disorders.

**Objective:**

This study aims to determine and compare the effectiveness of telepractice and the parent-implemented EMT+PE intervention on language and speech measures in toddlers with CP/L with usual interventions and determine the effectiveness maintenance of the intervention.

**Methods:**

A randomized controlled trial (RCT) will assess the efficacy of telepractice and the parent-implemented EMT+PE intervention in enhancing speech and language measures in toddlers with CP/L. Eligible participants will be randomly assigned to one of 2 groups: the conventional intervention group and the EMT+PE intervention group. Participants’ speech and language measures will be evaluated remotely by trained raters before and after the intervention and 2 months after the intervention. Parents of participants in the intervention group will receive 3 months of training in speech and language supportive strategies from trained therapists using telehealth fidelity scales. Parents of participants in the control group will receive the conventional speech and language intervention by cleft team therapists. Study outcomes will include language variables (mean length of utterance) and speech production variables (percent correct consonants).

**Results:**

The protocol was approved by the Research Ethics Committee of the University of Social Welfare and Rehabilitation Sciences in February 2022. The selection process of participants, as well as training therapists and raters, commenced in January 2022, the therapy and follow-up period ended in June 2023, and pre- and postintervention assessments have been conducted. Data analysis is ongoing, and we expect to publish our results by the summer of 2024. Funding is yet to be received.

**Conclusions:**

The results of this study may help us develop a speech and language intervention with a different delivery model for toddlers with CP/L, and the cleft team care can use these results in service delivery. Consistent with our hypothesis, speech and language measures are expected to improve.

**International Registered Report Identifier (IRRID):**

DERR1-10.2196/54426

## Introduction

### Background

Cleft palate with or without cleft lip (CP/L) frequently leads to difficulties with speech and language [1-5]. Previous studies have reported that children with CP/L have limited consonant inventory, which leads to decreased expressive vocabularies [6-8]. Thus, trained and experienced speech-language pathologists (SLPs) in cleft and craniofacial teams often provide speech-language therapy services for these children. Team-based cleft care for this population follows particular intervention protocols. However, due to limited cleft teams in some countries or regions, especially in low-income and transitional countries, and the long distance between the cleft teams and families’ residences, many of these children are unable to receive appropriate speech-language therapy services. Parent training and telepractice therapy sessions could help fill this gap [9-12].

Training and coaching for parent-implemented interventions have been known as practical methods to prevent or improve speech-language disorders in children [9-14]. Parents are considered the first teachers of their children’s speech and language development since they spend considerable time with their children in a natural environment, which provides them many opportunities to teach their children through daily routines. Intervention approaches in speech-language pathology have highlighted the crucial role of parents in these approaches. Parents are trained to use supportive language and speech strategies while interacting with their children [14]. Training strategies parents use in a child’s environment also facilitate the transfer of therapy from clinical settings to the child’s natural environment [15-17]. A systematic review of 18 studies [18] analyzing parent-implemented interventions revealed that parents who received training increased their responsiveness, usage of language models, and rate of communication with their children—in addition to the use of language-supportive strategies—had positive impacts on expressive language in preschool children with or without language impairments.

Language and motor intervention approaches have been used for children with CP/L. A systematic review [19] comparing the benefits of linguistic and phonological versus motor phonetics approaches revealed that although all the studies reported significant findings, they did not report a more effective intervention approach. Other studies [8] showed that both approaches improved consonant inventory; however, the linguistic and phonological approach was more effective in improving speech outcomes. Most of these studies have been conducted with children older than 4 years. It seems that the naturalistic-based approach is more effective in and applicable to younger children [20]. Enhanced Milieu Teaching (EMT) is one of the approaches of the naturalistic approach. It is a conversation-based naturalistic model for early language intervention that uses children’s interests and initiations as opportunities to model and encourage language use in daily routines [15]. EMT with Phonological Emphasis (EMT+PE) extends the prompting strategies of EMT to include Phonological Emphasis (PE). PE or speech recasts are a subset of recasts that target correct phonological production in response to the child’s incorrect productions [5,11,12,21]. Studies have reported positive effects of EMT+PE on language and speech outcomes including the percentage of correct consonants (PCC), consonant inventory, word production speed, compensatory errors, expressive vocabulary, and receptive language [5,21,22]. EMT+PE delivery methods are flexible for children with CP/L. Two pilot studies have demonstrated the effectiveness of the EMT+PE intervention via telepractice [11,12].

Telepractice refers to services delivered over long distances using videoconferencing or other technologies. Also, it is a service delivery model for different purposes in specific populations with communication disorders [13,23]. Telepractice is used to train parents in parent-implemented approaches [11,12,24]. Parent training via telepractice is implemented through different media such as videoconferences, YouTube videos, and web-based modules. The type of media for service delivery depends on the media facilities [24,25]. Previous research has indicated that treatment outcomes are comparable for both in-person and telepractice service delivery, and these methods are used as alternatives or as a hybrid approach [26].

The prevalence of cleft lip and palate (CLP) in Iran has been reported as 1.24 per 1000 births, with an incidence rate of 1 in 1000 births [27,28] There are 6 active cleft teams in Iran, all located in the center of major provinces. Iran, with a population of over 80 million individuals, of which 26% live in rural areas and 74% in cities, most families with a child with CLP are likely to face significant challenges in accessing team services or may not have the opportunity to use them due to their living conditions. According to various studies [26,29], telepractice service delivery can largely address the problems of health care services and provide timely access to services for children and families; therefore, our objective is to develop a protocol to evaluate the effect of a parent-based telepractice EMT+PE intervention on speech and language outcomes in Persian-speaking toddlers with nonsyndromic cleft palate, and compare this intervention to conventional, usual-care interventions.

### Aims

#### Primary Objectives

Our primary objective is to determine the effectiveness of a parent-based telepractice EMT+PE intervention on language and speech outcomes in toddlers with CP/L.

#### Secondary Objectives

Our secondary objectives are to compare the effectiveness of this parent-based telepractice EMT+PE intervention with usual-care interventions and determine the effectiveness of the intervention in the follow-up period.

## Methods

### Study Design

This is a single-blind, 2-arm, parallel randomized controlled trial (RCT).

### Study Population and Setting

**Figure 1:**
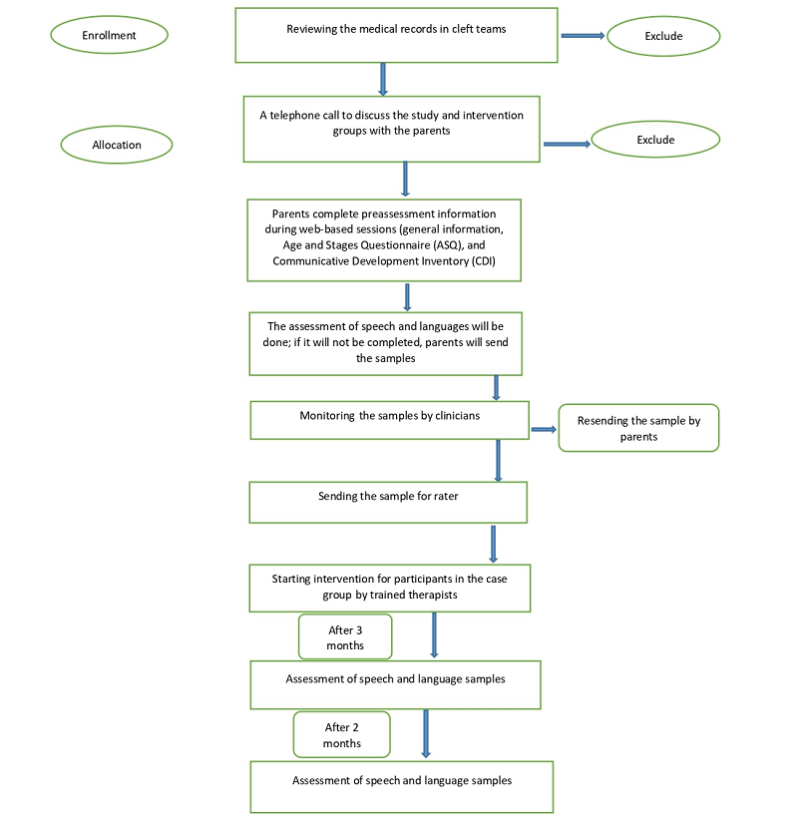
CONSORT (Consolidated Standards of Reporting Trials) flowchart showing the flow of participants in both groups.

The study will enroll 32 children with different types of oral cleft including unilateral cleft lip and palate, bilateral cleft lip and palate, and isolated cleft palate (Figure 1).

Participants will be recruited through local cleft palate teams in 3 major provinces (Esfahan, Tehran, and Shiraz), social media support groups, and local speech and language centers. All child-parent dyads will be required to meet the inclusion criteria.

### Inclusion and Exclusion Criteria

Eligible participants including children, their parents, and SLPs will be recruited through a rigorous review of the literature in this field [4,11,12,21].

#### Inclusion Criteria

##### Children

The inclusion criteria for children are as follows: (1) Persian-speaking children with CP/L aged between 18 and 36 months before the intervention, (2) lack of a diagnosis of a syndrome by a geneticist and by referring medical records to cleft care teams, (3) undergoing primary palate repair by the age of 15 months, (4) absence of sensorineural hearing loss or a sound field hearing threshold greater than 30 dB HL (decibels in hearing level), (5) preintervention assessment of typically developing children wherein scores on the Age and Stages Questionnaire in all domains (communication, fine motor, gross motor, personal and social, and problem-solving skills) are within the normal range (±1 SD from the cutoff points in each domain), (6) the child’s sufficient joint attention with the parent during the collection of play-based language samples, (7) at least 1 type of compensatory error throughout the speech sample, and (8) the child can produce at least 5 different words as measured using the Macarthur-Bates Communicative Development Inventory (MCDI).

##### Parents

The inclusion criteria for parents are as follows: (1) having literacy skills at least at the level of elementary school education, (2) being interested in participating in the assessment and telepractice training sessions, and (3) being able to use a cell phone containing apps that will be used in the intervention.

##### SLPs

The inclusion criteria for SLPs are as follows: (1) having at least 5 years of experience in providing treatment approaches for children with speech-language disorders, (2) not currently delivering EMT+PE and not previously trained in the interventional approach, and (3) having experience in treating children with various speech-language disorders via telepractice.

#### Exclusion Criteria

##### Children

Children will be excluded from the study if they (1) are bilingual or do not speak Persian, (2) have other dysmorphic features that affect speech (fistula in the palate), (3) have been receiving either therapy in the last 6 months or speech therapy in private clinics, or (4) are participating in another research study involving an intervention or multiple assessments.

##### Parents

Parents will be excluded from the study if they are (1) unable to properly implement speech- and language-supportive strategies or (2) unwilling to continue with the intervention process.

##### SLPs

SLPs will be excluded from the study if they are unable to provide weekly family therapy sessions.

### Randomization and Blinding

#### Overview

After preassessments, participants will be allocated to either of 2 intervention groups to eliminate selection bias and control for any extraneous variables: EMT+PE or the usual-care intervention. Block randomized trials will be conducted for each age group using the blockrand package in R software (version 4.1.3; The R Foundation). Randomization will be based on age (3 age groups) and the 2 interventions. The flow of participants through the study is illustrated using the CONSORT (Consolidated Standards of Reporting Trials) flowchart in Figure 1.

#### Blinding of Raters and Reliability of the Data

Trained and experienced raters who are blinded to the grouping of participants will transcribe audio and video samples of speech and language. The samples will be collected from the parents at home as they are interacting with their children using toys and pictures, using the Profiles of Early Expressive Phonological Skills (PEEPS) assessment.

### Intervention

#### EMT+PE Intervention

**Figure 2:**
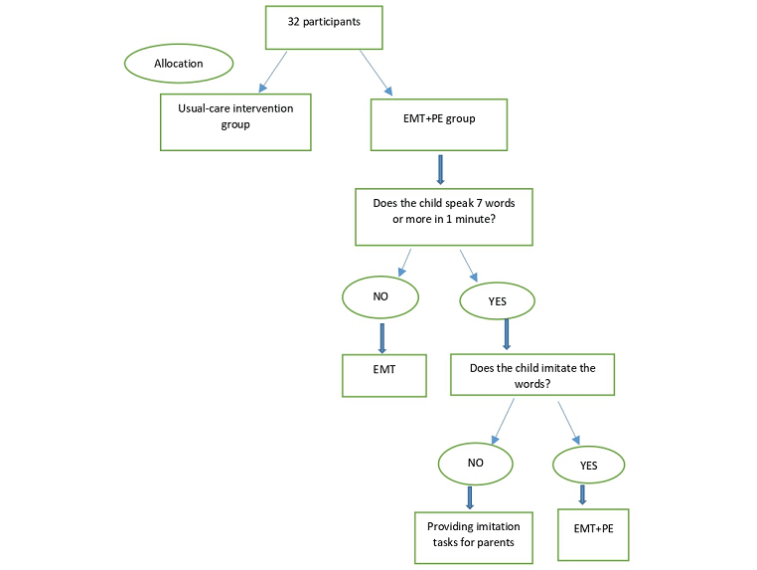
Principles of the EMT+PE (Enhanced Milieu Teaching with Phonological Emphasis) intervention. EMT: Enhanced Milieu Teaching.

The EMT+PE intervention consists of implementing supportive speech and language strategies by parents in their interaction with their children. All parents will be trained in accordance with the Teach-Model-Coach-Review (TMCR) instructional model that has been adapted for the telepractice environment [9,30]. The Teach component of the instructional approach involves introducing targeted supportive strategies. The Model component includes the implementation of strategies by a clinician when interacting with a child—we will implement this component by showing recorded video examples of strategies. The Coaching aspect entails parents using strategies during their interaction with their children. The Review component includes monitoring and evaluating the entire session and planning for the next session at the end of every session. EMT+PE treatment principles for the intervention group are illustrated in Figure 2.

#### Speech Target Selection

Speech targets for each child are determined by analyzing the errors produced in their speech sounds during the PEEPS assessment. A list of sample words will be created from the speech sound errors observed in the children’s PEEPS assessment. To select the target speech sounds, developmental norms and the frequency of speech sound errors related to cleft palate, including high-pressure and fricative consonants in words with the syllabic consonant-vowel-consonant-vowel structure, are taken into account. For each child, place, and manner of articulation, pre- and postvocalic voicing are considered. Once a participant can produce 7-10 words per minute, a speech recast strategy is integrated into the intervention process.

#### Usual-Care Intervention

Children assigned to the usual-care intervention (control) group will be assessed twice before and after the 3-month intervention. If they are not, they will be enrolled in cleft teams. They will receive speech and language therapy there in accordance with a specific protocol. Regular evaluations administrated every 3-6 months, are part of the usual speech and language intervention for children aged up to 3 years undergoing team care. Based on each child’s evaluation, early counseling is provided to parents in person or remotely. Parents are expected to apply the recommendations provided until the next visit. The EMT+PE will not be administered to participants in the control group throughout the experimental intervention period.

### Procedure

#### Therapist Training

We will recruit 3 eligible SLPs based on the study inclusion criteria. The process of implementing the protocol will be explained to them by the first author; if they are responsible for implementing it, they will participate in training sessions. They will receive training regarding the study methodology, participants, EMT+PE intervention, strategies, and fidelity of implementation for therapists and parents. SLPs will receive at least 3 in-person training sessions in accordance with the TMCR instructional model. For the Teach component, the research team will introduce speech and language strategies and their rationale. For the Model component, video examples related to the implementation of strategies by professional therapists in interacting with a child with a language disorder will be shown [12]. In the Coach component, therapists will implement strategies through role-playing. Thereafter, for the Review component, all training sessions will be monitored by researchers and therapists, and questions related to session content will be discussed and answered. For the competency-based assessment according to telehealth fidelity scales, each therapist will train a parent of a child with CP/L on the telegram platform so that the groups will consist of a therapist, a parent, and the main researcher. Then, the therapist will train the parent in at least 1 language strategy during offline and web-based sessions, and the parent will send a video of using the trained strategy when interacting with her child. Two independent experts will observe the skills-based assessment sessions and rate the therapists’ performance using telehealth fidelity scales.

#### Parent Training

Parents will receive training on specific strategies and apply them while interacting with their children at home. The competency-based assessment of parents will be defined using fidelity scales that are based on the trained strategies. The therapist will connect with parents twice a week for 3 months during the intervention course. The training sessions will be held in both offline and web-based modes. The therapist will share the material with the parents during offline sessions to teach them the strategies. For the model component, the therapist will send video examples of the strategies to the parents. Parents will be provided sufficient time to study the documents and view the video examples of the strategies. At the next stage of the administration process, the therapist will schedule a video call with the parents and they will discuss the content of the documents and video examples that would be sent, and talk about how to apply the strategies when interacting with their child. After the discussion, the parents will submit a video that shows them implementing the taught strategy. The therapist will review the videos sent. Next, the weaknesses and strengths of the video will be described on the basis of a fidelity checklist through a video call. Trainees will learn 7 supportive speech and language strategies that are arranged hierarchically from easy to difficult, including environment arrangement, match turns, modeling, prompting, time delay, expansion, and recasting [12,22]. The environment arrangement strategy aims to enhance the child’s engagement with the physical setup by selecting, arranging, and managing materials. In the matched turns strategy, parents are encouraged to match their turns of conversation with those of their child and carry out language mapping, in which parents are encouraged to mirror their child’s actions during play and incorporate language into the shared actions. Modeling involves a verbal model by the parent in which the child is encouraged to imitate. In the prompting strategy, the parent encourages the child to use an utterance at the target level in a conversational interaction. In the time delay strategy, parents encourage their child to promote initiation by providing nonverbal cues rather than relying on verbal models and commands; this includes assistance, pause in routine action (when the parent is interacting with their child), visual selection, and inadequate portion (ie, parents must ensure that the toys or equipment that they provide to their children are complete and ready to use; for example, if a doll is in a box, the child may require assistance in unboxing it and starting to play with it). In the expansion strategy, the parents repeat their child’s utterance and add a word or phrase to complete its utterance. Speech recasting involves repeating a child’s phrases with modified grammar or speech production (or both).

#### Rater Training

The child’s speech and language samples are transcribed by 4 experienced and trained raters who are blinded to the child’s group allocation (intervention or control). Before transcribing the samples, they will be provided instructions on how to rate the samples. To assess the reliability of the data, 25% of each rater’s samples will be scored by an independent expert. In case of inadequate agreement (ie, <85%) differences will be identified, and the samples will be reevaluated.

### Fidelity Assessment

#### Fidelity of Implementation for Therapists

Therapist fidelity will be assessed using an observational checklist with Likert scales. This checklist is designed in accordance with the components of the TMCR instructional model and the telepractice environment (Kaiser Telehealth Fidelity Scale). The checklist includes 5 sections: teach, model, coach, review, and overall interaction. Each section has several statements to measure implementation quality by the therapist. After training sessions with the therapists and before starting the intervention, they will be assessed using the fidelity checklist while delivering the intervention to a parent. The reliability of each therapist will be assessed (Multimedia Appendix 1).

#### Fidelity of Implementation for Parents

Parents’ ability to implement the trained EMT+PE strategies with procedural fidelity will be determined through formative visual analysis based on established learning criteria. To ensure fidelity, therapists and an independent expert will assess parents’ use of each strategy during 8 monthly sessions, using a checklist. The criteria for the strategies are as follows: environment arrangement (80%), language mapping (75%), match turns (75%), expansions (50%), modeling (40%), child’s target level (50%), prompt (80%), delay (80%), and speech recast (40%). The reliability of parents’ fidelity will be measured during the intervention [12,13] (Multimedia Appendix 2).

### Data Collection

In total, 32 children with CP/L in different provinces of Iran (Isfahan, Tehran, and Shiraz) who meet the primary inclusion criteria will be recruited. Next, the first author will call the parents and explain the process and the study’s terms of acceptance. Then, she will schedule a web-based session to complete the assessment. Parents will complete demographic information forms followed by the Age and Stages Questionnaire for their children’s developmental screening. Parents will collect speech and language samples before and after the intervention for their child in the naturalistic environment (Multimedia Appendix 3).

### Primary Outcomes

#### Speech Outcome Measures

The early phonological development of Persian-speaking monolingual children with CP/L is assessed using the Persian adaptation of the PEEPS assessment, which focuses on early acquired vocabulary [31]. The PEEPS assessment consists of 60 words that are expected to be expressed by Persian-speaking monolingual children aged 18-36 months. When administering the PEEPS assessment, an assessor first presents a picture or toy to the child as a target word; at this stage, the child is expected to respond independently. If the child does not respond, the assessor asks a question that requires the child to cue a specific word (eg, “What is this?”). If the child fails to respond again, the assessor utters a sentence that the child must complete with the target word (eg, “The baby has...”). Finally, if the child cannot produce the target word despite these cues, the assessor provides a direct command (eg, “Say...”). If the child still cannot produce the target word in response to the final command, it is marked as having provided no response.

In this study, the PEEPS assessment will be administered to assess consonant inventory based on the word-initial and word-final consonants. Also, the accuracy of speech production will be assessed by determining the total PCC, PCC stops, whole structure match, and whole-word accuracy match by an expert during a web-based session [32,33]. The evaluation session will be recorded. If the child does not participate actively in the web-based PEEPS assessment session, the parent will follow the assessor’s instructions to record their child’s responses to the speech sample and send them to the assessor.

The second version of MCDI provides parents with a categorized vocabulary list and asks them to indicate whether their children can express each word or phrase. The scale has 2 sections and assesses 680 expressive vocabulary items in 22 semantic categories among young children aged 16-30 months [34].

#### Language Outcome Measures

During the assessment, parents will engage in play sessions with their children using various toys. A 15-minute language sample will be collected remotely to determine the child’s number of different words, number of total words, and language complexity measured by mean length utterances. The assessment session will be video recorded. If the child does not engage during the web-based assessment session to collect language samples, the parent will follow the assessor’s instructions for recording the child’s responses and send the video recording to the assessor.

### Secondary Outcomes

Speech intelligibility will be measured using the Intelligibility in Context Scale, which is a parent-reported social validity tool [35]. The intelligibility rating is determined by the average response on a 5-point rating scale over 7 questions [36].

>Parents will be required to complete a postintervention satisfaction survey. The questionnaire aims to measure parent satisfaction with the telepractice EMT+PE intervention and parent training sessions (Multimedia Appendix 3).

### Statistical Analysis

#### Analysis

The analysis will be carried out using descriptive and analytical statistics. Descriptive statistics appropriate for summarizing demographic information and assessment data will be used. Preintervention differences between groups will also be estimated. The data analysis will be carried out using the SPSS (version 19; IBM Corp). The study’s outcomes will be presented as an estimation of the difference between groups, with a 95% CI and its associated *P* value. The statistical significance level will be set at α=.05*.* We will use analysis of covariance, an efficient statistical test that provides impartial estimations of an intervention’s effects, assuming random treatment assignment; this test is more powerful than other alternative statistical strategies. We will also carry out a paired samples *t* test, which compares the means of 2 variables within a single group; it estimates the differences between the values of 2 variables for each case and tests whether their average is significantly different from 0. Additionally, data from all randomized participants will be included in the intention-to-treat analysis.

#### Intended Sample Size

Based on comparable studies with a 95% confidence level, 80% power of the test, variance of 0.0036 units, and an accuracy (improvement) of 0.06 units in the PCC, each group requires a maximum sample size of 16 participants.

### Ethical Considerations

Participants’ safety risk will not be increased by participating in this trial. Each participant’s information will be stored under a unique number, and the coded information will be appropriately stored. When the research is completed, the results will be presented at scientific conferences or published in scientific journals, and the identities of study participants will remain confidential. The parents will be required to complete the informed consent form before participating in the study, and both the researcher and the participant must sign the document, with a copy provided to the participant. This study has been approved by the Research Ethics Committee of the University of Social Welfare and Rehabilitation Sciences, Iran (IR.USWR.REC.1400.310).

## Results

The protocol was approved by the Research Ethics Committee of the University of Social Welfare and Rehabilitation Sciences in February 2022. The selection process of participants, training therapists, and raters, commenced in January 2022, the therapy and follow-up periods ended in June 2023, and pre- and postintervention assessments have been conducted. Data analysis is ongoing, and we expect to publish our results by the summer of 2024. Funding is yet to be obtained.

## Discussion

This study is the first RCT with follow-up, which aims to develop a protocol to evaluate the effect of parent-based EMT+PE through telepractice in Persian-speaking toddlers with nonsyndromic cleft palate.

In this study, the impact of the interventions will be evaluated by language and speech outcomes as primary outcome measures. Language outcomes include a vocabulary inventory, collected using the MCDI, mean length utterances, number of total words, and number of different words, collected through language samples and during the parent-child interaction. Speech outcomes including PCC, consonant inventory, whole-word accuracy match, and whole structure match will be collected during the PEEPS assessment. The effect of interventions will be examined for 12 weeks, with an additional 8-week follow-up period for the EMT+PE group to exclude any temporary effects that may have limited clinical value. Our results will provide information about the efficacy of the parent-implemented EMT+PE telepractice intervention in enhancing speech skills while also promoting speech production and language measures and its stability over a prolonged period. In addition, we will obtain parents’ satisfaction and score on the Intelligibility in Context Scale before and after delivering interventions as secondary outcome measures. Improvement of speech intelligibility is a long-term target of most speech therapy interventions.

The study’s limitations include its small sample size for each age group. To address the effects of such interventions, a larger-scale clinical trial is necessary. Additionally, the intervention and follow-up periods are limited in demonstrating effects. It is important to note that toddlers are in different age ranges and may require more time to develop their language and speech skills.

In summary, we anticipate demonstrable effects of the parent-implemented EMT+PE telepractice intervention on language and speech outcomes in toddlers with CP/L; these should specifically include not only an extended vocabulary inventory but also an improved consonant inventory and speech intelligibility. This study’s results may help develop a specific intervention with a different delivery model for toddlers. Parent training through telepractice has multiple benefits for families, including providing therapy for children with speech-language disorders, reduced costs, unrestricted access to therapy services for people living in rural areas, and saving time and expenses [24-26]; therefore, cleft care teams can harness our results in service delivery.

## Appendix

Multimedia Appendix 1 Telehealth Fidelity Scales.

Multimedia Appendix 2 EMT+PE Fidelity Rating Form. EMT+PE: Enhanced Milieu Teaching with Phonological Emphasis.

Multimedia Appendix 3 Study timeline.

## Abbreviations

CLP: cleft lip and palate
CONSORT: Consolidated Standards of Reporting Trials
CP/L: cleft palate with or without cleft lip
dB HL: decibels in hearing level
EMT: Enhanced Milieu Teaching
EMT+PE: Enhanced Milieu Teaching with Phonological Emphasis
MCDI: Macarthur-Bates Communicative Development Inventory
PCC: percentage of correct consonants
PE: Phonological Emphasis
PEEPS: Profiles Of Early Expressive Phonological Skills
RCT: randomized controlled trial
SLP: speech-language pathologist
TMCR: Teach-Model-Coach-Review

## Data Availability

The research team will retain all the data obtained from the study. In compliance with applicable legal and statutory obligations, the coordinating principal investigator will archive all study-related documents for 5 years upon completion of the study.
