# Supplementary material for: Telepractice Parent Training of Enhanced Milieu Teaching With Phonological Emphasis (EMT+PE) For Persian-Speaking Toddlers With Nonsyndromic Cleft Palate: Protocol for a Randomized Controlled Trial
Source: JMIR Res Protoc. 2024 Apr 19;13:e54426. doi: 10.2196/54426 (PMC11069098; doi:10.2196/54426)
Supplement: Multimedia Appendix 1 [file resprot_v13i1e54426_app1.pdf]

Telehealth Fidelity Scales

(Developed By Ann P. Kaiser)

teach	ITEMS	SCORING	SUGGESTIONS
**T.1 Session Focused**	The therapist does not select a session focus based on the probe or selects an inappropriate focus (according to the decision tree).	0	
	The therapist selects an appropriate focus based on the probe but does not discuss the decision with the caregiver**.**	1	
	Based on the probe, the therapist appropriately decides the session focus and discusses the decision with the caregiver**.**	2	
**T.2 Strategy Review**	The therapist does not review the target strategy.	0	
	The therapist gives a cursory review that misses one or more components (explanation, rationale, and/or examples).	**1**	
	The therapist gives a thorough review including an explanation, rationale, and examples.<br>OR<br>If in the review phase, gives rationale and examples only	**2**	
**T.3. Strategies Examples**	The therapist does not show the caregiver video clips of the strategy NOR does she give a verbal example**.**	0	

	The therapist shows the caregiver a video clip of the strategy but does not encourage the caregiver to reflect on what they observed??.	1	
	The therapist shows the caregiver a video clip of the strategies and encourages the caregiver to reflect on what they observed .<br>OR<br>If the parent has already seen multiple clips or is in the review phase, give verbal examples.	2	
**T.4. Caregiver Understanding**	the therapist misses clear signs of caregiver misunderstanding and/or confusion about the strategy and does not make any attempt to adapt the instruction to clarify.	0	
	The therapist does not do anything to gauge the caregiver's understanding. By the end of the teach portion, it is not known if the caregiver grasps the strategy concepts.	1	
	The therapist gauges the caregiver's understanding and adapts instruction if the caregiver misunderstands or appears confused. By	2	

	the end of the teach portion, the caregiver has demonstrated an understanding of the strategy concepts.		
**T.5 Session Plan**	The therapist does not discuss the plan for session activities with the caregiver.	0	
	The therapist informs the caregiver of the plan for session activities without the caregiver's input.	1	
	The therapist jointly plans the session activities with the caregiver by either proposing an idea and asking for input or by asking the caregiver for an idea or preference.	2	
**LANGUAGE MODELING**	**ITEMS**	**SCORING**	**SUGGESTIONS**
**1. Therapist Use of Strategies**	The therapist never models examples of target-level language.	0	
	The therapist rarely (1-3x) models examples of target-level language.	1	
	The therapist sometimes (4-6x) models examples of target-level language.	2	
	The therapist often (7-9x) models examples of target-level language.	3	
	The therapist very often (≥10x) models)	4	

	examples of target-level language.		
2. Therapist Narration of Strategy	The therapist never highlights his/her use of target-level language.	0	
	The therapist rarely (1x) highlights his/her use of target-level language.	1	
	The therapist at least occasionally (≥2x) highlights his/her use of target-level language.	2	
**Coach**	**ITEMS**	**SCORING**	SUGGESTIONS
**C.1. Specificity Of Feedback**	The therapist's feedback is rarely or never (~0-25%) specific (i.e., nearly always vague)	**0**	
	The therapist's feedback is occasionally (~25-75%) specific.	1	
	The therapist's feedback is often (~75-100%) specific	2	
**C.2. Succinctness Of Feedback**	The therapist's feedback is often or always (~75-100%) wordy (i.e., rarely succinct)	0	
	The therapist's feedback is occasionally (~25-75%) wordy.	1	
	The therapist's feedback is rarely or never (~0-25%) wordy (i.e., nearly always succinct).	2	
**C.3. Pacing of Feedback**	The therapist's feedback is very poorly paced. This	0	

	includes providing feedback only at the beginning or end of the interaction.		
	The therapist's feedback is somewhat poorly paced. This includes providing feedback primarily at the beginning or end of the interaction and/or providing too infrequent feedback.	1	
	The therapist's feedback is well-paced throughout the interaction.	2	
**C.4. Salience of feedback**	The therapist never explains the rationale for a strategy based on the child's behavior.	**0**	
	The therapist rarely (1x) provides feedback about the caregiver's use of the strategy of the day.	**1**	
	The therapist at least occasionally (≥2x) provides feedback about the caregiver's use of the strategy of the day.	**2**	
	The therapist was unable to provide feedback about the chosen strategy.	**N/A**	
**C.5. Feedback On Rationale**	The therapist never explains the rationale for a strategy based on the child's behavior.	**0**	
	The therapist rarely (1x) explains the	**1**	

	rationale for a strategy based on the child's behavior.		
	The therapist at least occasionally (≥2x) explains the rationale for a strategy based on the child's behavior.	2	
**C.6.Feedback on The Impact Of Strategies**	The therapist never highlights the outcome of the caregiver's strategy use on the child's behavior.	0	
	The therapist rarely (1x) highlights the outcome of the caregiver's strategy used on the child's behavior.	1	
	The therapist at least occasionally (≥2x) highlights the outcome of the caregiver's strategy use on the child's behavior.	2	
**C.7. Constructiveness Of Feedback**	The therapist provides solely positive or solely constructive feedback.	0	
	The therapist provides an inappropriate ratio of positive versus corrective feedback based on the caregiver.	1	
	The therapist provides an appropriate balance of positive and constructive	2	

	feedback based on the caregiver.		
C.8. Caregiver Support	A) The therapist provides significantly too little support for the caregiver. This includes not helping when the caregiver is struggling.<br>B) The therapist provides significantly too much support. This includes giving excessive support despite caregiver competence.	**0**	
	A) The therapist provides somewhat too little support for the caregiver .<br>B) The therapist provides somewhat too much support for the caregiver.	**1**	
	The therapist provides an appropriate amount of either physical or verbal support for the caregiver.	**2**	
**REVIEW**	**ITEMS**	**SCORING**	**SUGGESTIONS**
R.1. Caregiver Reflection	The therapist is dismissive of the caregiver's attempts at reflection. This may include responding negatively to the caregiver's reflection or changing the topic when the caregiver attempts to reflect.	**0**	
	The therapist does not encourage the caregiver's reflection.	1	

	This includes not asking a probing question or not responding to the caregiver's spontaneous reflection with further reflective feedback.		
	The therapist actively encourages the caregiver to engage in a reflective discussion. This includes probing the caregiver to reflect and/or responding to their spontaneous reflection with further reflective feedback.	2	
**R.2.Caregiver Evaluation**	A) The therapist does not discuss or provide feedback about the caregiver's use of the strategies B) The therapist's feedback is negative or condescending.	0	
	•The therapist discusses how the caregiver used the strategies before encouraging the caregiver's reflection. •AND/OR doesn't link caregiver and child behavior •AND/OR feedback is only general or cursory.	1	
	•The therapist discusses how the caregiver used the strategies after the reflection	2	

	•AND notes a connection between caregiver and child behavior.<br>•AND provides helpful, specific feedback.		
**R.3. Home Carry Over**	The therapist does not discuss ways the caregiver can use the strategies during the week.	0	
	The therapist gives instructions to the caregiver about how they can use strategies throughout the week but does not discuss the caregiver	1	
	The therapist solicits the caregiver's perspectives and ideas about appropriate and helpful ways to carry over strategies throughout the week.	2	
**Overall interaction**	**ITEMS**	SCORING	SUGGESTIONS
**I.1. Ease Of Interaction**	The therapist appears uncomfortable or standoffish. The therapist may avoid or deter the caregiver's attempts to engage in discussion.	0	
	The therapist appears relatively at ease. While the therapist does not avoid or actively deter the caregiver's attempts to engage	1	

	in discussion, she does not make an effort to encourage discussion or ensure that the caregiver is at ease.		
	The therapist appears at ease throughout the interaction and readily engages in discussion and conversation with the caregiver. The therapist makes efforts to ensure that the caregiver is also at ease.	2	
**I.2. Positive Tone**	The therapist often appears disinterested, cold, and/or critical.	0	
	The therapist's tone and effect are primarily neutral. She does not appear to especially enjoy the visit or interacting with the caregiver/child.	1	
	The therapist appears to enjoy the visit, including showing warmth and humor. The therapist smiles frequently.	2	
**I.3. Respect of Family**	The therapist makes one or more comments that minimize the caregiver's experiences, preferences, beliefs, or lifestyle. The therapist may make "should/shouldn't"	0	

	comments regarding parenting/lifestyle choices.		
	The therapist does not make any judgmental or minimizing comments, but not does not actively demonstrate respect and appreciation for the caregiver's experiences and perspective.	1	
	The therapist demonstrates understanding, respect, and appreciation toward the caregiver. The therapist appears interested in the caregiver's perspective.	2	
