# Supplementary material for: Telepractice Parent Training of Enhanced Milieu Teaching With Phonological Emphasis (EMT+PE) For Persian-Speaking Toddlers With Nonsyndromic Cleft Palate: Protocol for a Randomized Controlled Trial
Source: JMIR Res Protoc. 2024 Apr 19;13:e54426. doi: 10.2196/54426 (PMC11069098; doi:10.2196/54426)
Supplement: Multimedia Appendix 2 [file resprot_v13i1e54426_app2.pdf]

EMT+PE Fidelity Rating Form

**Name:**	**reviewer:**				**video session:**
**Item**	**scoring**				***suggestions***
	0	1	2	3	
The environment provides physical boundaries and contains- age-appropriate toys (Environmental arrangement)					
Materials can be used to elicit multiple types of conversation and play (e.g., books, block with trucks, group times) (Environmental arrangement)					
The parent is responsive to the child's interest (i.e., joins in activities, is positive and energetic, is physically accessible, and is at the child's level). (Match turns or language mapping)					
Time delays are used when the child requests nonverbally or with minimal verbalizations. (Assistance, pause in routine, visual choice, and inadequate portion)					
Models include the child's targets. (Models, speech recasting)					
Provide a question or a model with" say X" if the child does not respond to the initial Time delay (models, prompting)					
Praise all correct requests, responses, and behaviors. (Matched turns)					
Uses choice questions during the session to elicit child targets. (prompting)					
Adult language, including expansions, does not exceed 2-3 words longer than the child's MLU). (expansions)					
Uses open-ended questions (i.e.," Tell me what you want?", "What do you want to do?"). (prompting)					
Expansions preserve as much of the child's utterance as possible. (expansions)					
The adults emphasize the target sound in the word the child produced incorrectly when recasting. (Speech recasting)					

EMT+PE Fidelity Rating Form

Name:	reviewer:				video session:
**Item**	**scoring**				***suggestions***
	0	1	2	3	
overall rating of EMT+PE strategies throughout the video					

0=intervention strategy is not present; 1: intervention strategy needs practice; 2: intervention strategy is inconsistent; 3: intervention strategy is excellent.
