# Supplementary material for: Telepractice Parent Training of Enhanced Milieu Teaching With Phonological Emphasis (EMT+PE) For Persian-Speaking Toddlers With Nonsyndromic Cleft Palate: Protocol for a Randomized Controlled Trial
Source: JMIR Res Protoc. 2024 Apr 19;13:e54426. doi: 10.2196/54426 (PMC11069098; doi:10.2196/54426)
Supplement: Multimedia Appendix 3 [file resprot_v13i1e54426_app3.docx]

Study Timeline

|  | Screening | Baseline assessment | Intervention period  (3 months) | End of Therapy | 2-month follow-up  (for the treatment group) | End of study |
| --- | --- | --- | --- | --- | --- | --- |
| Time point | three weeks | T1 |  | T2 | T3 |  |
| **ENROLMENT:**  Eligibility screen  Informed consent  Allocation |  |  |  |  |  |  |
|  | **×** |  |  |  |  |  |
|  | **×** | **×** |  |  |  |  |
|  |  |  |  |  |  |  |
| **INTERVENTION:**  EMT+PE  Intervention as usual |  |  |  |  |  |  |
|  |  |  |  |  |  |  |
|  |  |  |  |  |  |  |
| **ASSESSMENTS:**  Demographics  ASQ  ICS  MCDI |  |  |  |  |  |  |
|  |  | **×** |  |  |  |  |
|  |  | **×** |  |  |  |  |
|  |  | **×** |  | **×** | **×** |  |
|  |  | **×** |  | **×** | **×** |  |
| Speech sample (ACM, WSM, CI, PCC, PCC STOPS) |  | **×** |  | **×** | **×** |  |
| Language sample (MLU, NTW, NDW) |  | **×** |  | **×** | **×** |  |
| Training of raters |  | **×** |  |  |  |  |
| Fidelity of parents |  |  |  |  |  |  |
| Training of therapists | **×** |  |  |  |  |  |
| Fidelity of therapist |  | **×** |  |  |  |  |
| Recording EMT+PE intervention sessions |  |  |  |  |  |  |
| Satisfaction form |  |  |  |  |  | **×** |

EMT+PE: Enhanced Milieu Teaching+ Phonological Emphasis.

ASQ; Age and Stages Questionnaire.

ICS: Intelligibility in Context Scale.

MCDI: MacArthur-Bates Communicative Development Inventory.

ACM: whole word Accuracy Match.

WSM: Whole Structure Match.

CI: Consonant Inventory.

PCC: Percentage of Consonant Correct.

MLU: Mean Length of Utterance.

NTW: Number of Total Words.

NDT: Number of Different Words.
